# Microstructural Understanding of Flow Accelerated Corrosion of SA106B Carbon Steel in High-Temperature Water with Different Flow Velocities

**DOI:** 10.3390/ma16113981

**Published:** 2023-05-26

**Authors:** Ying Hu, Long Xin, Chang Hong, Yongming Han, Yonghao Lu

**Affiliations:** National Center for Materials Service Safety, University of Science and Technology Beijing, Beijing 100083, China; huying2017@aliyun.com (Y.H.);

**Keywords:** flow-accelerated corrosion, microstructure, localized corrosion, flow velocity, carbon steel

## Abstract

All light or heavy water reactors fabricated with carbon steels suffer from flow-accelerated corrosion (FAC). The FAC degradation of SA106B with different flow velocities was investigated in terms of microstructure. As flow velocity increased, the major corrosion type changed from general corrosion to localized corrosion. Severe localized corrosion occurred in the pearlite zone, which can be the prior location for generating pits. After normalizing, the improvement in microstructure homogeneity reduced the oxidation kinetics and lowered cracking sensitivity, causing a decrease in FAC rates of 33.28%, 22.47%, 22.15%, and 17.53% at flow velocity of 0 m/s, 1.63 m/s, 2.99 m/s, and 4.34 m/s, respectively. Additionally, localized corrosion tendency was decreased by reducing the micro-galvanic effect and tensile stresses in oxide film. The maximum localized corrosion rate decreased by 21.7%, 13.5%, 13.8%, and 25.4% at flow velocity of 0 m/s, 1.63 m/s, 2.99 m/s, and 4.34 m/s, respectively.

## 1. Introduction

As one of the material degradation processes, flow-accelerated corrosion (FAC) can happen in flowing water and result in wall thinning of pipes or components in nuclear power plants [1,2]. All light or heavy water reactors built with carbon steel suffer from FAC. A pipe rupture accident may occasionally occur due to the thinning of the pipe wall. The wall thinning of pipes has been managerially controlled by means of non-destructive inspection and evaluation of remaining lifetime [3,4]. Therefore, understanding the factors influencing FAC is of great importance in order to predict FAC rate.

Generally, the material microstructure, hydrodynamic variables, pH, water temperature, and dissolved oxygen concentration are the main factors influencing FAC [5]. On the side of the fluid dynamic factor, FAC rate is restricted by mass transfer for influencing the kinetic of degradation. The soluble species diffused from the oxide to the bulk fluid is facilitated by the mass transfer. Moreover, the flow velocity must be highly considered on account of the diffusion being the driving force of FAC [6,7]. The diffusion coefficient is mainly related to the concentration gradient of soluble species, which is inversely related to the thickness of the boundary layer. The thickness of the boundary layer in dynamic water is much thinner than that in static water, which leads to a greater corrosion rate in dynamic water. Therefore, the difference between them could reach 10–25 times [6], and the corrosion rate increased linearly with the increase in the flow velocity [8]. Moreover, the entire corrosion matches with a loss of bulk material in the solution and not with a thick layer of duplex oxide [9]. Under circulating solution conditions, in the low-flow velocity zone, the mass transfer of soluble iron species at the oxide surface dominates the FAC rate. Furthermore, the FAC rate increases with decreasing concentration. In the intermediate-flow velocity zone, the anodic reaction at the metal surface is much smaller than the mass transfer coefficient. The FAC rate changes to be controlled by the electrochemical corrosion for the bulk metal. In high-flow velocity zones, the surface shear stress is significantly increased so that the oxide film is broken or mechanically removed. Then, an erosion process starts and is mixed with FAC. In this case, the degradation is regarded as erosion–corrosion [10].

From the viewpoint of microstructure, the distribution and constitution of the phases play an important role in the corrosion performance. Under certain conditions, the uniform distribution of the phases benefit corrosion resistance [11]. The aggregation of pearlite can be found to be a banded structure, which is an internal defect in the steel [12,13]. A banded structure may destroy the uniformity of the steel. Furthermore, the accumulation of lamellar cementite in pearlite is regarded as the major reason for the inferior corrosion resistance of ferrite–pearlite steel in the long run of exposed corrosion [14,15]. As an important second phase in ferrite–pearlite steel, the distribution, morphology, and size of the cementite are closely associated with the anti-corrosion performance. Because of the potential difference between ferrite and cementite, severe localized corrosion often occurs in flowing solution, resulting in the accelerated corrosion of ferrite–pearlite [16,17]. In addition, localized corrosion always comes into being at the position of local damaged film if high flow velocity restrains the regeneration of beneficial oxide film [18,19]. Obviously, the deterioration of the ferrite–pearlite steel for pipelines is dominated by uniform corrosion and localized corrosion.

Heat treatment is an effective way to modify microstructure features of engineering steels. Correspondingly, in order to relieve the effect of the banded structure, the accumulation of cementite, and the localized corrosion, a normalizing heat treatment is employed to obtain uniformly dispersed ferrite and pearlite in this study. Moreover, we mainly focus on the related microstructure evolution during FAC with different flow velocities and offer direct evidence for the material degradation. Moreover, the comparative study of as-received ferrite–pearlite steels and normalized steels in the simulated secondary side water environment of nuclear power plants is meticulously reported and analyzed, with the establishment of physical models for material failure.

## 2. Materials and Methods

### 2.1. Material Preparation

The as-received samples were machined from the SA106B pipeline used for a pressurized water reactor nuclear power plant in China. The chemical composition of SA106B is shown in Table 1.

### 2.2. FAC Tests

**Figure 1:**
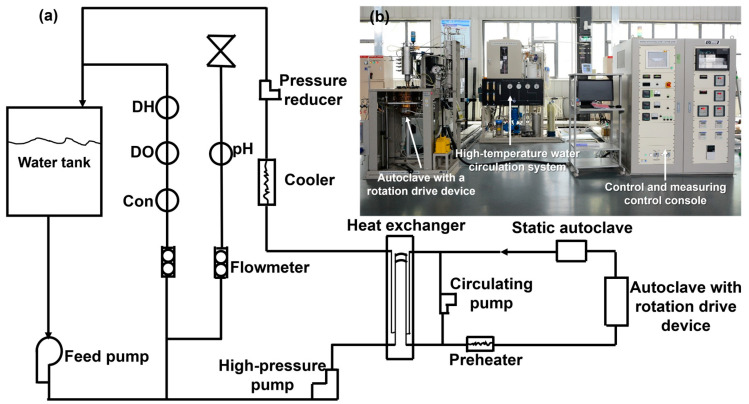
(**a**) The schematic of the high-temperature water FAC test facility and (**b**) its real picture in our lab.

The FAC tests were performed by the subcritical water environment rotating-cylinder electrode testing system (SW-RETS) in our lab. Figure 1a shows the schematic of the FAC test facility. It contains the high-temperature water circulation system, an autoclave with a rotation drive device, a static autoclave, control and measuring control console, and data-acquisition system. Figure 1b shows the real scene for the working FAC test facility. The temperature, pressure, pH, dissolved oxygen (DO), dissolved hydrogen (DH), and conductivity can be controlled and measured by the high-temperature water circulation system. High pure nitrogen gas was pumped into the loop continuously to control the DO concentration. The pH value was modified by ammonia water. The autoclave with a rotation drive device and the static autoclave are manufactured by SUS 316L with 3.5 L (Toshin Kogyo Co., Ltd., Tokyo, Japan). The type of rotation drive device is the servo motor control, by which the rotational speed can be outputted within 20–2000 rpm.

**Figure 2:**
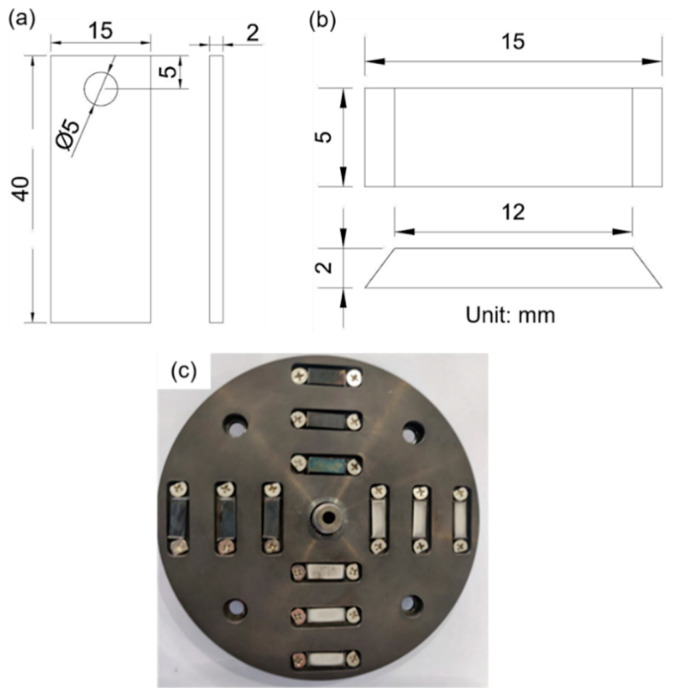
The specimen size in the (**a**) static autoclave and (**b**) dynamic autoclave with the rotation drive device. (**c**) The rotating disc with mounted samples.

Figure 2a shows the specimen size in the static autoclave. The specimens were insulated and assembled to a holder. Then, the holder with specimens was fixed in the static autoclave. This is regarded as the flow velocity of 0 m/s. Figure 2b shows the specimen size in the autoclave with a rotation drive device. As can be seen, the specimen has a trapezoid shape. After mechanical polishing, the specimens were mounted into the rotating disc (Figure 2c). The trapezoid specimens were insulated from the fixture by the zirconia sheets. Only the test surface (area: 12 mm × 5 mm) was exposed to the high-temperature water. The testing rotational speed was chosen as 865 rpm. Correspondingly, the linear velocity of the inner, middle, and outer circle could be calculated as 1.63 m/s, 2.99, m/s, and 4.34 m/s. Each circle had four specimens.

In the secondary circuit pipes, the pH value of high-temperature water was controlled at 9.7, avoiding the occurrence of FAC caused by a low pH value [20] and preventing the stress corrosion cracking of the steam generator caused by a high pH value [21]. At the same time, the DO value was also controlled at below 10 ppb to avoid under-deposit corrosion caused by the deposition of oxide on the surface of the heat-exchange tubes [22,23]. Based on the actual operating temperature of the secondary circuit [6], and in order to study the FAC mechanism at extreme temperature, the test temperature was selected as 290 °C. Thus, the FAC test conditions and water chemistry parameters in this study can be summarized in Table 2.

### 2.3. Analyses

According to ASTM G1-03, the oxide film was removed. Then, the weight loss was calculated by the difference value between the weight before the FAC test and the weight after removing the oxide film. The weight was measured by an electronic balance with an accuracy of 0.01 mg. Then, the FAC rate (ν) could be calculated by Equation (1) [24].

(1) $$Ν=\frac{87,600\Delta W}{\rho \mathrm{St}}$$

where ν is the FAC corrosion rate (mm/y), ΔW is the weight loss (g), ρ is the density of steel (g/cm^3^), generally considered as 7.8 g/cm^3^, S is the superficial area of the sample (cm^2^), and t is the duration time (h).

The specimens after FAC tests were firstly machined by wire-electrode cutting perpendicular to the direction of the linear speed. Secondly, the cross-section was ground by emery paper and mechanically polished with silica suspension to be a mirror surface.

The volume fraction of the corrosion pit area was measured by Image J software (Version 1.50i). For a sample in one state, at least five scanning electron microscopy (SEM) pictures of sample were imported, which could be pieced together to form the entire FAC surface morphology of the sample. Thus, the mean volume fraction of the corrosion pit area with error bars from those SEM pictures can accurately display the surface features of a sample in one state.

The SA106B specimens were etched in 4% nitric acid alcohol solution to show the microstructure, which was observed via optical microscopy (OM, Zeiss). Nanomeasurer software was used to quantify the grain size and the lamellar spacing. After FAC tests, the corrosion surfaces and the cross-sections were characterized via SEM (Zeiss Merlin compact). The corrosion pit profiles were measured by laser scanning confocal microscopy (LSCM, OLYMPUS-OLS4000). The Raman mapping analyses for the oxides in the cross-section were detected by the high-resolution in-situ SEM-Raman system (Zeiss) with an excitation wavelength of 633 nm. Transmission electron microscopy (TEM) specimen was fabricated using a dual-beam focused ion beam (FIB) instrument (FEI Helios Nanolab 600i) via an in-situ lift-out method. Carbon was deposited as a protective layer preventing beam damage. TEM (Talos F200X G2) with a “Super X” energy dispersive spectroscopy (EDS) detector system was used to observe the cross-sectional microstructure and elemental distribution. High-resolution transmission electron microscopy (HRTEM) was performed. Then, Fast Fourier Transformation (FFT) and inverse FFT (IFFT) diffractograms were used to analyze the high-resolution images. In scanning transmission electron microscope (STEM), high-angle annular dark field (HAADF) images were obtained with EDS elemental mapping analyses.

## 3. Results

**Figure 3:**
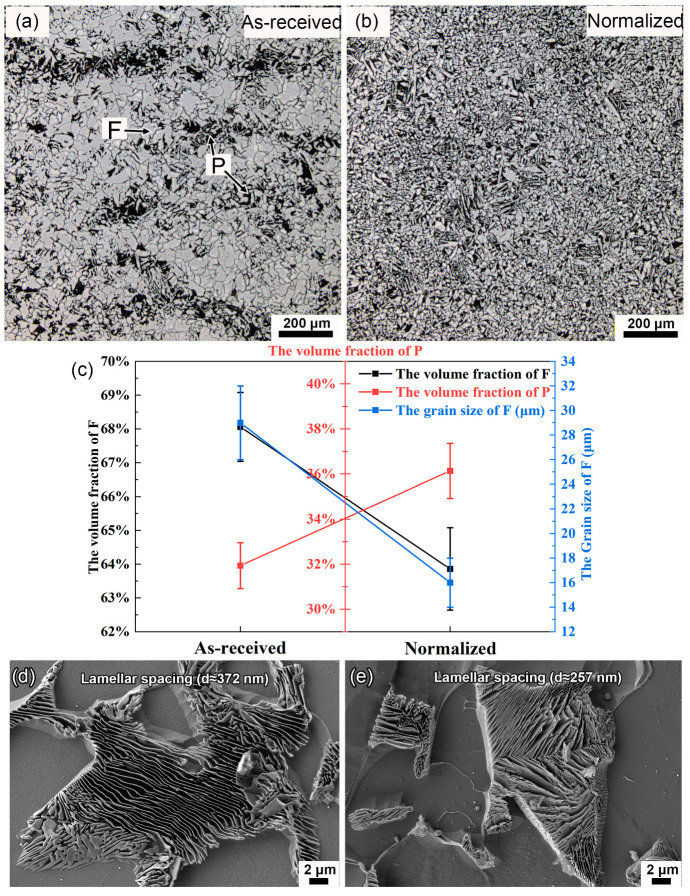
OM images of (**a**) as-received and (**b**) normalized samples. The bright and dark regions represent ferrite (F) and pearlite (P), respectively. (**c**) The volume fractions of F and P in as-received and normalized samples. SEM images of pearlite lamellae in (**d**) as-received and (**e**) normalized samples.

As shown in Figure 3a, the typical microstructure of as-received sample consists of equiaxed proeutectoid ferrite (F) embedded by pearlite (P) islands with eutectoid ferrite ($F^{\prime }$) and cementite (Fe_3_C). Banded structure can be observed in the steel. In order to eliminate banded structure, the normalizing heat treatment was applied. Firstly, the furnace was heated up to 920 °C. Then, the samples were put into it and held for 20 min. Finally, the samples were cooled in air. The microstructure of normalized sample also consisted of F and P, which were evenly distributed. Meanwhile, the banded structure disappeared (Figure 3b). The volume fraction of F decreased from 68.06% to 63.86%, while the volume fraction of P increased from 31.94% to 36.14%. The grain size of F decreased from 29 μm to 16 μm (Figure 3c). Figure 3d,e show the scanning electron microscopy (SEM) images of P in as-received and normalized samples. The close-up observation finds that pearlite lamellae were not always straight, but accompanied by local bending and misfitting. The lamellar spacing was obviously refined from ~372 nm to ~257 nm after normalizing.

**Figure 4:**
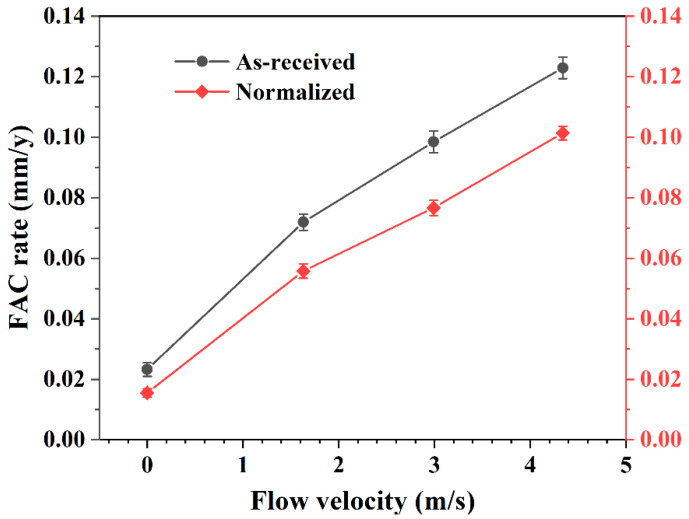
FAC rates of as-received and normalized samples under different flow velocities. The error bars are calculated by the standard deviation of at least three measurements from each sample.

Figure 4 shows the FAC rates of as-received and normalized samples under different flow velocities. Compared to static conditions, the FAC rate under dynamic conditions was much larger. Furthermore, the FAC rate increased linearly with the increasing flow velocity under dynamic conditions. For as-received samples, the FAC rate increased from 0.072 mm/y to 0.123 mm/y as the flow velocity increased from 1.63 m/s to 4.34 m/s. Compared with the as-received samples, the FAC rate of normalized samples was obviously smaller. It increased from 0.056 mm/y to 0.101 mm/y as the flow velocity increased from 1.63 m/s to 4.34 m/s.

**Figure 5:**
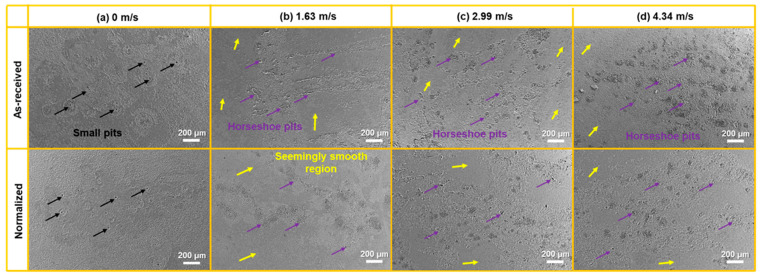
Low-magnification SEM images of the corrosion surfaces at the velocities of (**a**) 0 m/s, (**b**) 1.63 m/s, (**c**) 2.99 m/s, and (**d**) 4.34 m/s in as-received and normalized samples. The small pits, horseshoe pits, and seemingly smooth region are marked by black, purple, and yellow arrows, respectively.

Significant differences in the SEM surface morphologies at different flow velocities in as-received and normalized samples are clearly visible in Figure 5. After being exposed in the static autoclave (0 m/s), corrosion was general with some small pits (marked by black arrow) which can be observed in Figure 5a. The number of small pits in normalized sample seems to be smaller than that in as-received sample. This is in agreement with the small weight loss in the normalized sample. As the flow velocity increased to 1.63 m/s (Figure 5b), the surface morphology obviously changed to be a combination of horseshoe pits (marked by purple arrow) and the seemingly smooth region (marked by yellow arrow). Some horseshoe pits were isolated while others gathered together to form a strip parallel to the flow direction. As the flow velocity further increased (Figure 5c,d), the area of horseshoe pits increased, while the area of the seemingly smooth region decreased.

**Figure 6:**
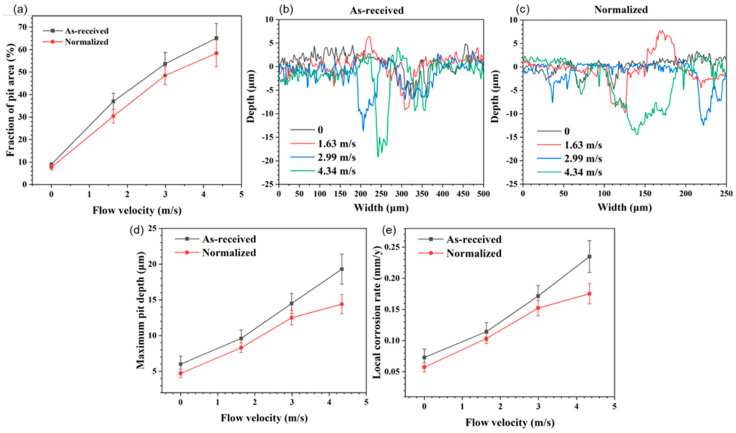
(**a**) The volume fraction of the localized corrosion pit area at different flow velocities in as-received and normalized samples. The profile of the cross-section at different flow velocities in (**b**) as-received and (**c**) normalized samples. (**d**) The maximum pit depth in as-received and normalized samples calculated from (**b**,**c**). (**e**) The corresponding local corrosion rate calculated from (**d**). The error bars are calculated by the standard deviation of at least five measurements.

Figure 6a shows the volume fraction of the localized corrosion pit area at different flow velocities in as-received and normalized samples. As can be seen, the volume fraction of small pit was low at 0 m/s. It decreased slightly after normalizing. As the flow velocity increased from 0 m/s to 4.34 m/s, the volume fraction of pit area increased from 8.97% to 65.14% in as-received samples, and from 7.68% to 58.37% in normalized samples. The difference value between the as-received and normalized samples gradually increased with an increase in the flow velocity. Figure 6b,c show the profile of the cross-section at different flow velocities in as-received and normalized samples, respectively. It can be found that the surface roughness of as-received sample was larger than that of normalized sample. Further, the maximum pit depth shown in Figure 6d was measured based on Figure 6b,c. As can be seen, the maximum pit depth was low at 0 m/s. It decreased slightly after normalizing. As the flow velocity increased from 0 m/s to 4.34 m/s, the maximum pit depth increased from ~6.0 μm to ~19.3 μm in as-received samples, and from ~4.7 μm to ~14.4 μm in normalized samples. The maximum pit depth in normalized samples was lower than in as-received samples. Figure 6e shows the local corrosion rate according to Figure 6d. The local corrosion rate in dynamic high-temperature water was larger than in static high-temperature water. It increased linearly as the flow velocity rose.

**Figure 7:**
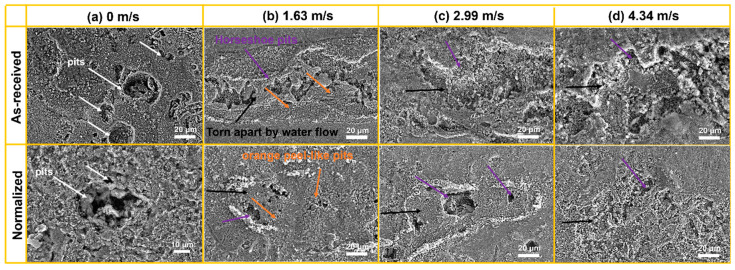
The magnified SEM images of the pit region at the velocities of (**a**) 0 m/s, (**b**) 1.63 m/s, (**c**) 2.99 m/s, and (**d**) 4.34 m/s in as-received and normalized samples. The pits, horseshoe pits, orange peel-like pits, and area torn apart by water flow are marked by white, purple, yellow, and black arrows, respectively.

Figure 7 shows the magnified SEM images of the pit region at different flow velocities in as-received and normalized samples. At 0 m/s, the pits with different size could be found in as-received and normalized samples (Figure 7a). As the flow velocity increased to 1.63 m/s, orange-peel-like or horseshoe-like pits appeared. The pits in as-received sample seemed to be torn apart by the water flow. The accumulation of local oxide particles could be found in the normalized sample (Figure 7b). As the flow velocity further increased to 2.99 m/s, the pit size obviously increased. However, the pit size in normalized sample was smaller (Figure 7c). As the flow velocity increased to 4.34 m/s, the pit size further increased. The pit size in normalized sample was still smaller than in as-received sample (Figure 7d).

**Figure 8:**
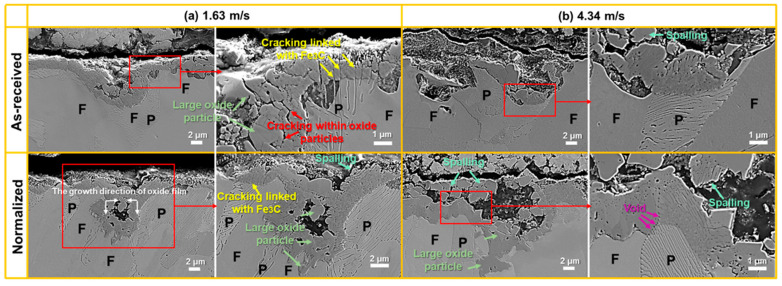
The SEM images of the cross-section of the localized corrosion pits at the velocities of (**a**) 1.63 m/s and (**b**) 4.34 m/s in as-received and normalized samples.

Figure 8a,b, respectively, show the SEM images of the cross-section of the localized corrosion pits at the velocities of 1.63 m/s and 4.34 m/s in as-received and normalized samples. It can be clearly seen that pits were formed inside the oxide film. Two types of oxides were formed around the pits. One was a large oxide particle with a size of 1–3 μm, which could be found at the outermost surface and near the ferrite (F). Cracking occurs within the oxide particles, which results in the delamination of the oxide film. The oxidation front always extends into the F with the shape of large oxide particle. The other was the collection of small-size oxides which were next to the pearlite (P). Furthermore, cracking is always linked with the cementite (Fe_3_C), which can also result in the delamination of oxide film. A void could be found at the interface of the oxide film and the P, which may be the origin of the cracking, as shown in Figure 8b. Additionally, the oxide film grew along the vertical and horizontal directions. As shown in Figure 8a, a complete oxide film was formed at the bottom of the pit at 1.63 m/s, which may effectively hinder the ion transport between the solution and the steel. After normalizing, the close of the pit seemed to be faster. The oxides may fully fill the pit and restrict the growth of localized corrosion. The growth in the oxide film decreased the pit diameter. As shown in Figure 8b, when the flow velocity increased to 4.34 m/s, the oxide film inside the pit found it difficult to grow along with the aggravation of localized corrosion. The normalized specimens had thicker oxides compared to the as-received specimens.

**Figure 9:**
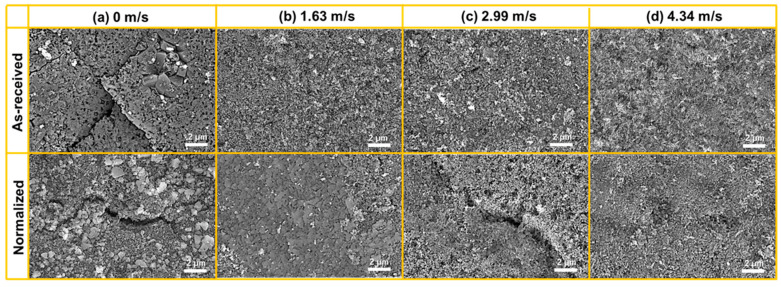
The magnified SEM images of the seemingly smooth region at the velocities of (**a**) 0 m/s, (**b**) 1.63 m/s, (**c**) 2.99 m/s, and (**d**) 4.34 m/s in as-received and normalized samples.

Figure 9 shows the magnified SEM images of the seemingly smooth region at different flow velocities in as-received and normalized samples. Actually, the seemingly smooth region (SSR) at 0 m/s consisted of the surface oxide film in which the cracking with random direction can be seen (Figure 7a). As the flow velocity increased to 1.63 m/s, the size of oxide particle obviously decreased. However, the aggregation of small-size oxides was still obvious in normalized sample (Figure 7b). As the flow velocity further increased to 2.99 m/s and 4.34 m/s, the size of oxide particles was almost unchanged (Figure 7c,d). In addition to a few cracks in normalized sample at 2.99 m/s, cracks rarely appeared from 1.63 m/s to 4.34 m/s.

**Figure 10:**
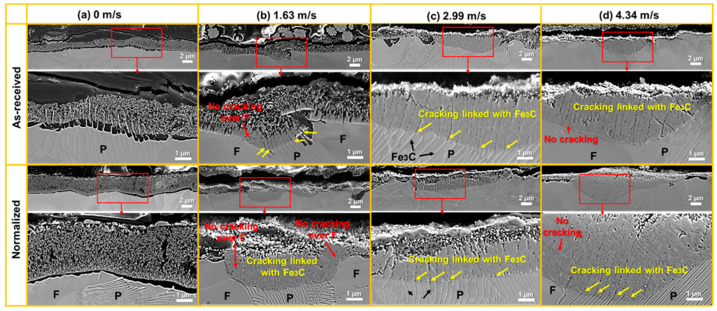
The SEM images of the cross-section of the seemingly smooth region at the velocities of (**a**) 0 m/s, (**b**) 1.63 m/s, (**c**) 2.99 m/s, and (**d**) 4.34 m/s in as-received and normalized samples.

Figure 10 shows the SEM images of the cross-section of the seemingly smooth region at different flow velocities in as-received and normalized samples. At 0 m/s (Figure 10a), the oxide film consisted of small-sized oxides with numerous pores. The morphology of the oxide was almost the same between as-received and normalized sample. The oxides above ferrite (F) and pearlite (P) had no difference. However, the thickness of the oxide film in normalized sample was larger than that of as-received sample. As the flow velocity increased to 1.63 m/s (Figure 10b), the thickness of the oxide film decreased. A salient phenomenon was that the oxidation above P was faster than that of F, which resulted in the formation of thicker oxide film. Cracking could be seen in the oxide film above P, which is linked with Fe_3_C (marked by yellow arrows). However, no cracking was found in the oxide film over F (marked by red arrows). The cracking width and length in as-received sample were larger than those of normalized sample. As the flow velocity increased to 2.99 m/s and 4.34 m/s (Figure 10c,d), the thickness of the oxide film further decreased. Cracking linked with Fe_3_C could also be found in the oxide film. The crack direction was not parallel to Fe_3_C, which may be due to the local bending and misfitting of Fe_3_C. There were almost no cracks in the oxide film over F. It is worth noting that cracking becomes discontinuous after normalizing.

**Figure 11:**
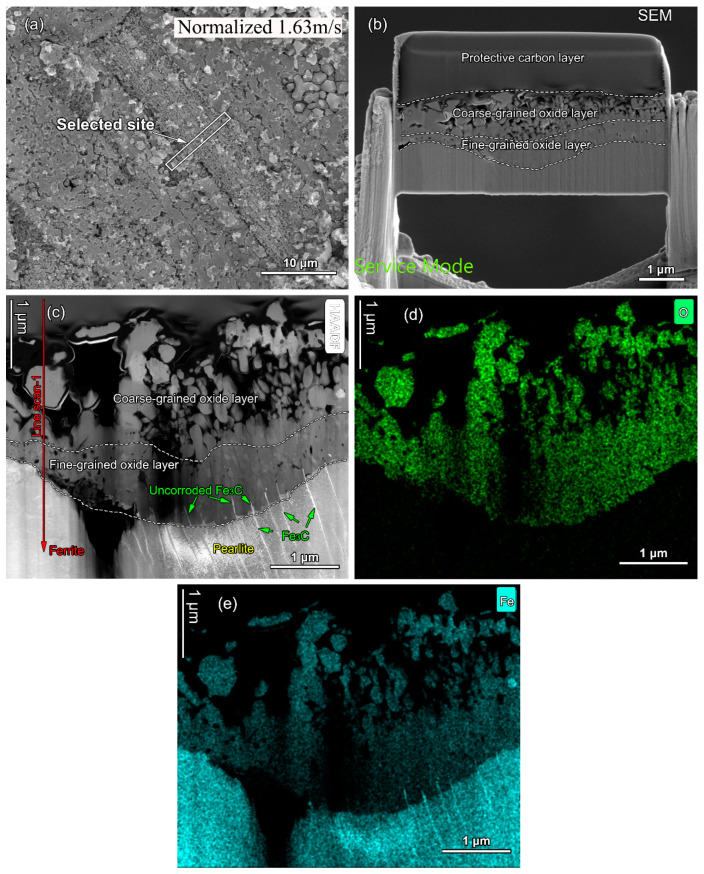
(**a**) The SEM image of the horseshoe pit region at a flow velocity of 1.63 m/s in normalized sample showing the selected site for TEM thin foil during the FIB lift-out process. (**b**) The cross-section SEM image of the thin foil. (**c**) The corresponding STEM-HAADF image and the EDX elemental mapping of (**d**) O and (**e**) Fe.

Figure 11a shows the SEM image of the horseshoe pit region at the flow velocity of 1.63 m/s in normalized sample showing the selected site for TEM thin foil during the FIB lift-out process. Figure 11b shows the cross-section SEM image of the thin foil. The protective carbon layer was just above the oxide film. The oxide film can be divided into the coarse-grained oxide layer and fine-grained oxide layer. Figure 11c–e shows the corresponding STEM-HAADF image and the EDX elemental mapping of O and Fe. Under STEM-HAADF imaging, the oxide film was dark while the bulk material was bright. Furthermore, the ferrite and pearlite can be properly distinguished due to the highlight of the cementite (Fe_3_C) in pearlite. It can be noted that there is uncorroded Fe_3_C in the oxide film, which is rich in Fe and O.

**Figure 12:**
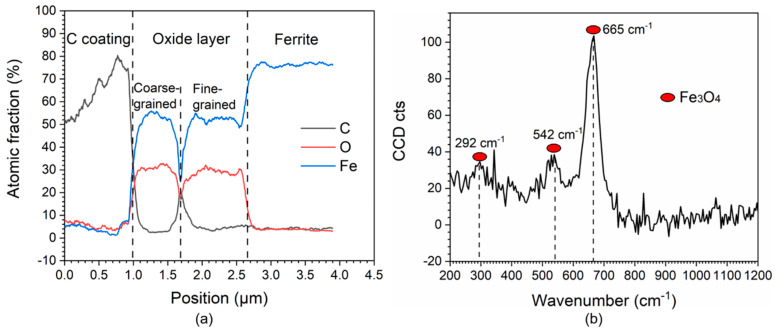
(**a**) The EDX line scanning analyses along line 1 in Figure 11c. (**b**) Raman mapping analyses for the oxide film.

Figure 12a shows the EDX line scanning analyses along line 1 in Figure 11c. It can be seen that the coarse-grained and fine-grained oxide layers had same the chemical elemental content with ~52 at. % Fe and ~30 at. % O. Figure 12b shows the Raman mapping analyses for the oxide film. Only one type of oxide Fe_3_O_4_ was detected, with one major peak at the wavenumber of 665 cm^−1^ and two minor peaks at the wavenumber of 292 cm^−1^ and 542 cm^−1^.

**Figure 13:**
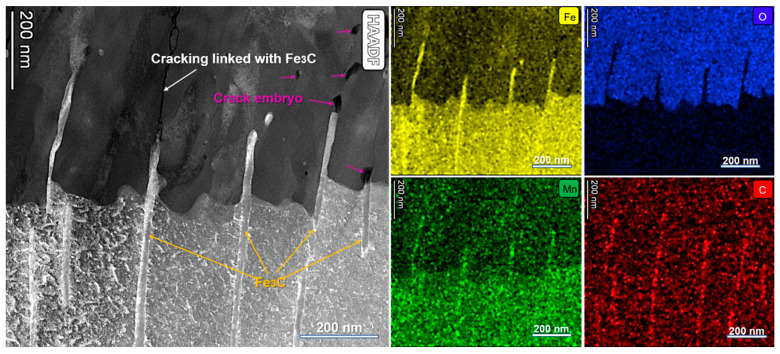
The magnified STEM-HAADF image and the EDX mapping of Fe, O, Mn, and C.

Figure 13 shows the magnified STEM-HAADF image and the EDX mapping of Fe, O, Mn, and C. As can be seen, the cementite (Fe_3_C) is rich in Fe, C, and Mn. Cracking linked with Fe_3_C could also be found under STEM-HAADF imaging (marked by white arrows). Furthermore, a crack embryo indicated by the darkest area (marked by pink arrow) appeared above the cementite and inside the oxide film. The darkest area signifies the lowest mass density under HAADF imaging, which indicates that the dark area should be thinner or porous [25]. Each dark piece is likely a crack embryo due to the low bonding strength of a porous layer, which can readily extend into a crack under further water flow.

**Figure 14:**
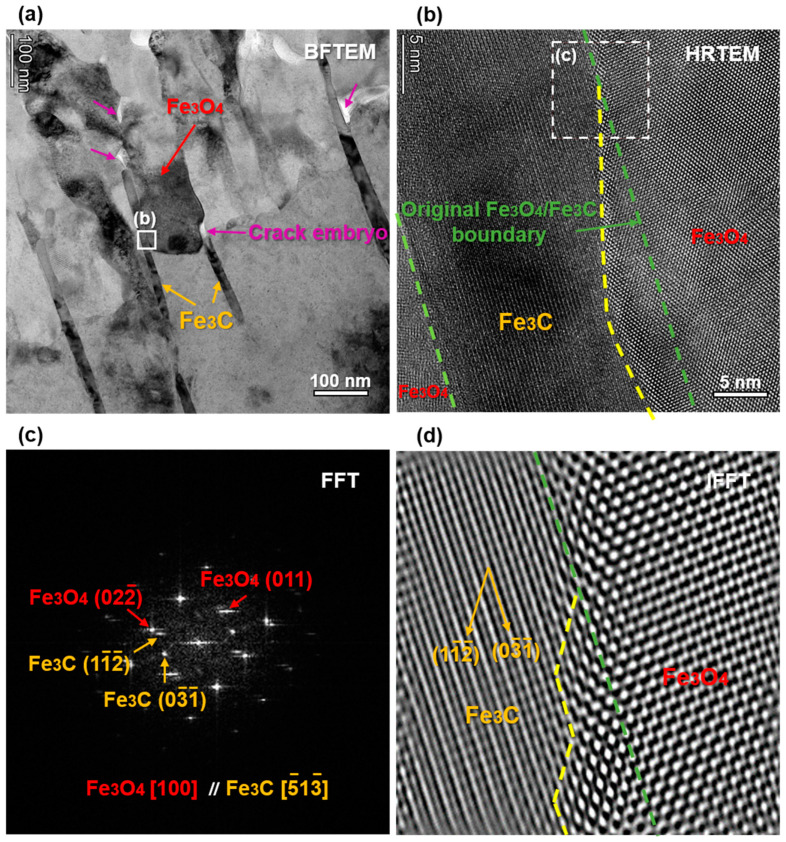
(**a**) The BFTEM image almost consistent with the HAADF image in Figure 13. (**b**) The HRTEM image taken from the rectangle in (**a**). (**c**) FFT and (**d**) IFFT images taken from the dash rectangle in (**b**).

Figure 14a shows the BFTEM image, which is almost consistent with the HAADF image in Figure 13. Crack embryos appeared bright under the BFTEM imaging. Additionally, crack embryo were located at the top and lateral of the Fe_3_C, the interface of Fe_3_O_4_/Fe_3_C, and the grain boundary of Fe_3_O_4_. Figure 14b shows the HRTEM image taken from the rectangle in Figure 14a. The original Fe_3_O_4_/Fe_3_C boundary can be clearly seen, and is marked by the green dash line. The fresh Fe_3_O_4_/Fe_3_C boundary is also clear, and is marked by the yellow dash line. The corrosion of Fe_3_C is likely preferential along a certain direction. In order to determine it, the FFT and IFFT analyses shown in Figure 14c,d were taken from the dash rectangle in Figure 14b. From Figure 14c, there was a relationship of Fe_3_O_4_{100}//Fe_3_C{$\overset{-}{5}1\overset{-}{3}$} with an incoherent boundary. The corrosion invasion into Fe_3_C took priority in the direction of Fe_3_C ($\overset{-}{1}1\overset{-}{2}$), as shown in Figure 14d.

## 4. Discussion

### 4.1. Influence of Microstructure on FAC

**Figure 15:**
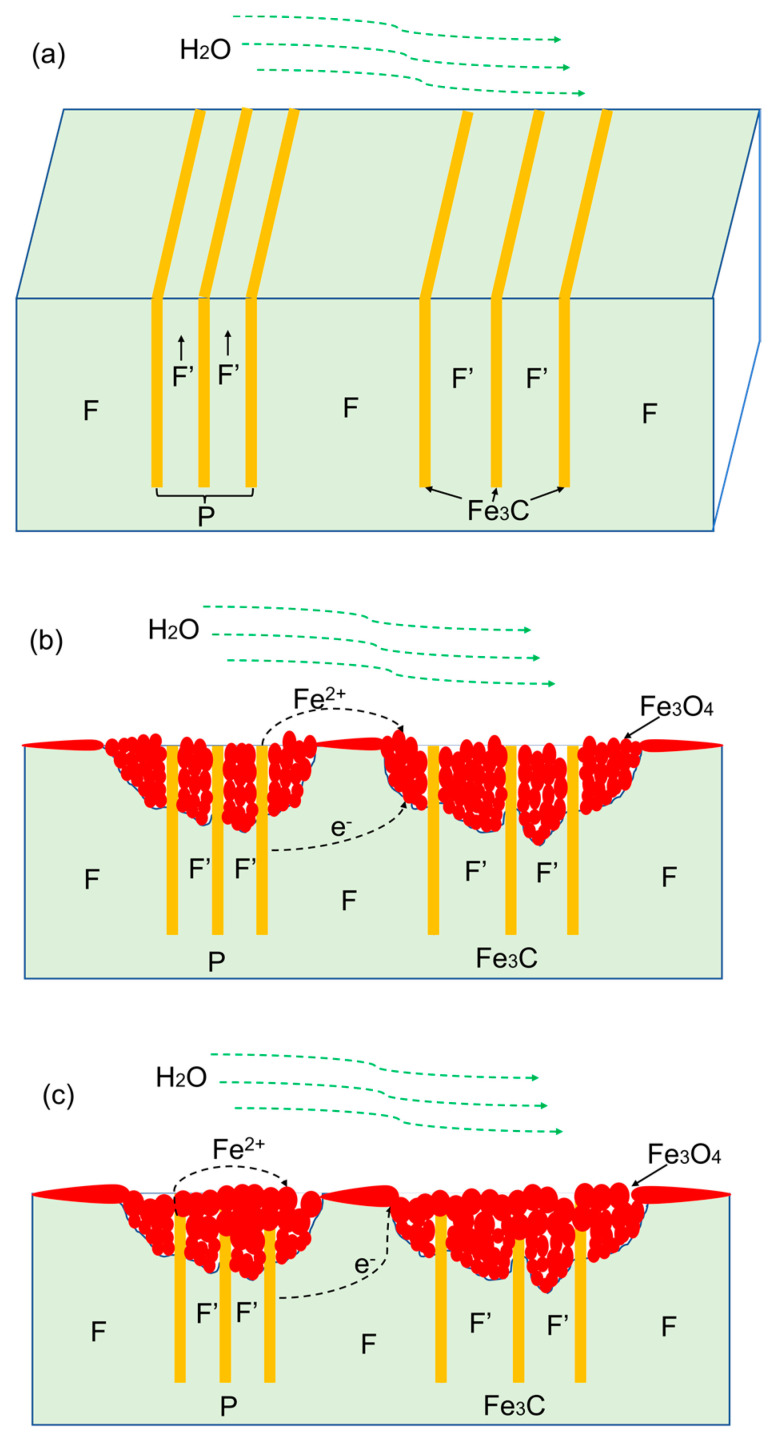
The schematic diagram of the evolution of localized corrosion related to the microstructure. (**a**) The microstructure of the steel, and the preferred corrosion for (**b**) $F^{\prime }$ and (**c**) Fe_3_C.

The microstructure played an important role in the initiation and propagation of FAC behavior, as elucidated in Figure 15.

For as-received SA106B carbon steel, the microstructure consisted of the polygonal proeutectoid ferrite (F) and the pearlite (P), which were composed of lamellar eutectoid ferrite ($F^{\prime }$) and lamellar cementite (Fe_3_C), alternately (Figure 3a and Figure 15a). When the samples were immersed in flowing HTHP water, the corrosion rate of the $F^{\prime }$ was faster than F (Figure 10b–d and Figure 11) due to the strong micro-galvanic effect between the lamellar $F^{\prime }$ and lamellar Fe_3_C [26,27,28], hence the corrosion rate on the cross-section was various. Only Fe_3_C (anode) was left behind after the $F^{\prime }$ (anode) dissolved preferentially. From this point, the P regions might be the preferred site for pit formation. As the immersion time increased, the fast-aggregating cathodic cementite resulted in an increase in cathode–anode area ratio, which intensified the micro-galvanic effect and further accelerated the dissolution of anodic $F^{\prime }$ [17,29]. Once the initial pits formed, the existence of remained cathodic Fe_3_C in the pits further accelerated the corrosion of ferrite in the pits, leading to the fast expansion of pits and severe localized corrosion ultimately, as shown in Figure 8. The initial pits could be used as the flow disturbances introducing the micro-turbulences around the anodic areas, which impaired the protective oxide layers formed at the ferrite zone and enhanced the local mass transport [30]. As a result, the ferrite in the pits was corroded more deeply, as shown in Figure 8.

As shown in Figure 15b, in high-temperature deaerated water, the only redox reaction of the ferrite in the iron/water system was reduction forming hydrogen. Iron was oxidized to produce Fe_3_O_4_ (Figure 12) based on the following Equation (2):

(2) $$3\mathrm{Fe}+4H_{2}O\to {\mathrm{Fe}}_{3}O_{4}+4H_{2}$$

Equation (2) is regarded as the combination of two simultaneous procedures [31]: the creation of iron hydroxide species by the direct oxidation of iron in oxygen-free water by Equation (3), and the initiation of magnetite through the Schikorr reaction (4).

(3) $$\mathrm{Fe}+2H_{2}O\to \mathrm{Fe}{\left(\mathrm{OH}\right)}_{2}+H_{2}$$

(4) $$3\mathrm{Fe}{\left(\mathrm{OH}\right)}_{2}\to {\mathrm{Fe}}_{3}O_{4}+H_{2}+2H_{2}O$$

As shown in Figure 15c, although the dissolution rate of Fe_3_C was lower than other phases, such as ferrite, the exposed Fe_3_C and the surrounding corrosion product film could sequentially form the condition of “small anode–large cathode” according to the following reaction (5) [32]. Furthermore, the reaction of Fe_3_C took priority in the direction of Fe_3_C ($\overset{-}{1}1\overset{-}{2}$) (Figure 14) having the largest crystal plane spacing, which was beneficial to the element diffusion.

(5) $${\mathrm{Fe}}_{3}C+2H_{2}O\to {\mathrm{Fe}}_{3}O_{4}+C+4H^{+}+4e^{-}$$

Crack embryos (Figure 10, Figure 13 and Figure 14) and cracking (Figure 8 and Figure 10) could be clearly seen at the interface of oxide/Fe_3_C, the oxide grain boundary, and the interface of ferrite/Fe_3_C. According to the research [33], the stresses were higher at the ferrite/Fe_3_C interface, where the oxidation was faster and more inclined to be cracking. The oxide grain boundary was always the site for the crack initiation and propagation [34].

For normalized SA106B carbon steel (Figure 3b), it was also composed of F and P. The F and P were evenly distributed on the whole surface without banded structure. Improvement in the microstructure homogeneity could relieve the localized corrosion tendency of ferrite–pearlite steel by reducing the corrosion acceleration caused by micro-galvanic effect [14,17,35]. The volume fraction of P was increased by 4.2%, while the lamellar spacing in P was reduced by 30.9% (Figure 3d,e). According to the study using electrochemical impedance spectroscopy for the steels with pearlite structure [36], the refinement of lamellar structure reduced the volume fraction of Fe_3_C available to act as cathodic sites, resulting in a decrease in the corrosion rate. After normalizing, an increase in the pearlite alignment in the substrate microstructure could weaken the tensile stresses in the oxide film [33], resulting in slower oxidation kinetics and lower cracking sensitivity, as shown in Figure 8 and Figure 10.

### 4.2. Influence of Flow Velocity on FAC

Under static conditions (0 m/s), general corrosion is the predominant type, as shown in Figure 10a. Under dynamic flowing conditions, however, localized corrosion becomes the major failure mode, as shown in Figure 6. The effect of flow velocity on FAC is usually conveyed by means of the wall shear stress imposed on the testing surface [24]. As shown in Figure 2c, the distance between the sample and the rotation axis is, respectively, 36, 66, and 96 mm. Thus, it can be assumed as a pipeline with 36, 66, and 96 mm in diameter, respectively. Hence, the wall shear stress (τ) in high-temperature water is able to be evaluated by Equation (6) [37]:

(6) $$\tau =\frac{1}{2}\rho_{w}{\mathrm{fv}}^{2}$$

where $\rho_{w}$, f, and v are the density of the dynamic aqueous phase (kg/m^3^), the Fanning friction factor, and the mean flow velocity (m/s), respectively. At present, $\rho_{w}$ is regarded as the water density, which is related to the temperature T (K) [38]:

(7) $$\rho_{w}=1152.3-0.5116T$$

Through the Equation (8), the Blasius friction factor for a smooth pipe wall [39], f, can be calculated with the Reynolds number (Re). Re is displayed as Equation (9).

(8) f=0.079Re−0.25

(9) Re=ρwVdμ

where d is the diameter of the pipeline (m). μ is the kinematic water viscosity (cp), which can be assessed according to the reference [40].

Accordingly, the formula for the wall shear stress associated with the density, kinematic viscosity, and flow velocity of high-temperature water is expressed as:

(10) $$\tau =0.0395\rho_{w}{}^{0.75}\mu^{0.25}v^{1.75}$$

**Figure 16:**
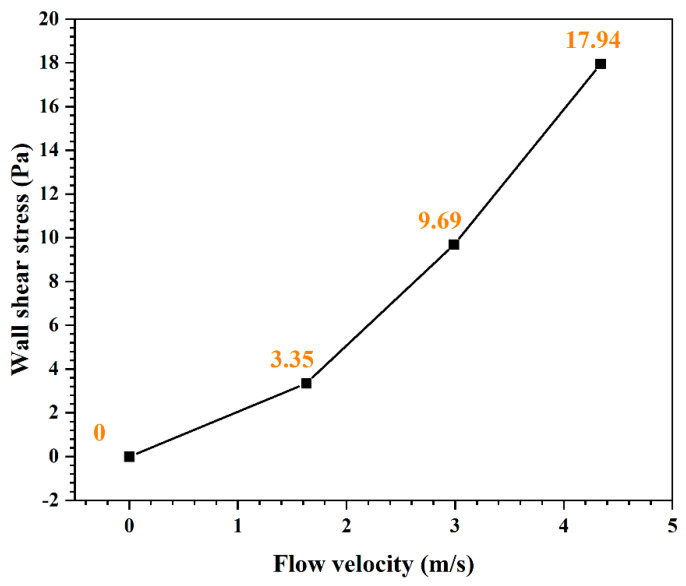
The change in wall shear stress of dynamic high-temperature water with flow velocity.

Figure 16 shows the change in wall shear stress with flow velocity, which is calculated by Equation (10). The wall shear stress increases with increasing flow velocity. When the flow velocity increases to 4.34 m/s, the wall shear stress grows to 17.94 Pa, which is much lower than the bonding strength (1–10^2^ MPa) between the film and the substrate [41]. Although wall shear stress is inadequate to lead to mechanical damage of complete Fe_3_O_4_ film, it still plays a significant role in the formation process of the oxide film. As the oxide film becomes denser, the internal stress simultaneously enhances with enlarging thickness of the oxide film [42], which could give rise to cracking in the film [43]. The spalling of oxides can be intensified by wall shear stress in the meantime, as shown in Figure 8. Additionally, the growth in Fe_3_O_4_ inside localized corrosion pits can also be influenced by the flow velocity. With increasing flow velocity, the vortex was readily created inside the pit to accelerate the mass transfer and prevent the formation of Fe_3_O_4_ film inside the pits. Then, severe localized corrosion was formed. The study [13] verified that 0.2 Pa of wall shear stress was already enough to exacerbate localized corrosion. Furthermore, the adjacent small pits could merge into a large pit under the impact of high flow velocity to worsen localized corrosion.

From Figure 8 and Figure 10, as the flow velocity increased, the thickness of the oxide film decreased. On the one hand, this may be attributed to spalling of the oxides by the increase in wall shear stress; on the other hand, the mass transfer process may play an important role in the decrease in the thickness of the oxide film. By Equation (11) [44] and Equation (12) [45], the mass transfer coefficient (K) increased with an increase in the flow velocity. Meanwhile, the formation of oxide film was controlled by the process of the deposition and dissolution of oxides, which were affected by the mass transfer process in boundary layer. When the flow velocity increased, the thickness of the boundary layer decreased. Thus, the ions from the dissolution of oxides and matrix diffused quicker. Therefore, the thickness of the oxide film was controlled by the flow velocity. After normalization, a denser oxide film formed on the surface of the SA106B after the FAC test, and the ions diffused out of the oxide film slowly, resulting in formation of thicker oxide film. By Equation (13) [46], the FAC rate was controlled by the thickness and porosity of the oxide film and the mass transfer coefficient (K), and the porosity of the oxide film was only affected by the temperature [5]. Thus, as the flow velocity increased, the thickness of the oxide film decreased and the mass transfer coefficient (K) increased, resulting in an increase in the FAC rate, and, after normalization, denser and thicker oxide film caused a lower FAC rate.

(11) $$K=\left(\frac{\tau }{{V\times \rho }_{w}}\right)S_{c}{}^{-\frac{2}{3}}$$

(12) $$S_{c}=\frac{\mu }{\rho_{w}\times D}$$

(13) $${\mathrm{FAC}}_{\mathrm{rate}}=\frac{\theta \left(C_{\mathrm{eq}}{-C}_{\infty }\right)}{\frac{1}{K^{*}}+0.5\left(\frac{\delta }{D}+1/K\right)}$$

where D is the diffusive coefficient of the ions (m^2^/s), θ is the porosity of the oxide film, δ is the thickness of the oxide film, $K^{*}$ is the kinetic constant of the reaction to form Fe(OH)_2_, $C_{\mathrm{eq}}$ is the iron ion concentration at equilibrium, and $C_{\infty }$ is the concentration of iron ions in the bulk solution, generally considered to be close to 0.

## 5. Conclusions

In this study, the FAC degradation of SA106B with different flow velocities was investigated in detail from the viewpoint of microstructure by using LCSM, SEM, and TEM. The following conclusions can be drawn:

➢ Flow velocity converted the major corrosion type in high-temperature water. The steel prevailingly went through uniform corrosion in static high-temperature water, while localized corrosion became dominant in dynamic water. The FAC rate in dynamic high-temperature water was much larger than static high-temperature water. Furthermore, the rate increased linearly as the flow velocity increased. After normalizing, the FAC rate was reduced by 33.28% under static conditions, while and 22.47%, 22.15%, and 17.53% at flow velocity of 1.63 m/s, 2.99 m/s, and 4.34 m/s, respectively.
➢ The faster the flow velocity, the more difficult it was to form a complete oxide film inside the pits, resulting in the aggravation of localized corrosion. With increasing flow velocity, the vortex was readily created even if the pit was small in size, with the effect of accelerating the mass transfer and preventing the formation of Fe_3_O_4_ film inside the pits, resulting in the severe localized corrosion. Meanwhile, enhanced wall shear stress could intensify the spalling of oxides and influence the growth in Fe_3_O_4_ inside localized corrosion pits. After normalizing, the close of the localized corrosion pits was faster. Oxides might fully fill the pit and restrain the growth of localized corrosion, and the maximum localized corrosion rate decreased by 21.7%, 13.5%, 13.8%, and 25.4% at flow velocity of 0 m/s, 1.63 m/s, 2.99 m/s, and 4.34 m/s, respectively.
➢ From the as-received steel with banded structure, severe localized corrosion occurred at the location of the pearlite, which could be the prior sites for pit initiation. The cementite acting as a cathode aggregated in the pits and further reinforced the effect micro-galvanic corrosion, resulting in the acceleration of the corrosion rate. Ultimately, the cementite was reacted during the corrosion invasion along the direction of Fe_3_C ($\overset{-}{1}1\overset{-}{2}$).
➢ From the normalized steel, uniformly dispersed ferrite and pearlite were obtained, along with the disappearance of banded structure. The improvement in microstructure homogeneity relieved the localized corrosion tendency by retarding accelerated corrosion through the micro-galvanic couple. The refinement of lamellar structure reduced the volume fraction of cementite available to act as cathodic sites, resulting in a decrease in the corrosion rate. The increase in pearlite alignment weakened the tensile stresses in the oxide film and led to slower oxidation kinetics and lower cracking sensitivity.

## Figures and Tables

**Table 1:** Chemical compositions of SA106B (wt.%).

Element								
Fe	C	Mn	P	S	Mo	Si	V	Cr
Bal.	0.21	0.53	0.011	0.008	0.01	0.26	0.01	0.02

**Table 2:** FAC test conditions and water chemistry parameters.

Rotational Speed	Duration	Temperature	Pressure	pH	DO
865 rpm	720 h	290 °C	10 MPa	9.7	<10 ppb

## Data Availability

The raw data required to reproduce these findings cannot be shared at this time as the data also form part of an ongoing study.
